# Characterizing Autism Traits in Toddlers with Down Syndrome: Preliminary Associations with Language, Executive Functioning, and Other Developmental Domains

**DOI:** 10.3390/bs16010039

**Published:** 2025-12-24

**Authors:** Tiffany Chavers Edgar, Claudia Schabes, Marianne Elmquist, Miriam Kornelis, Lizbeth Finestack, Audra Sterling

**Affiliations:** 1 Waisman Center, University of Wisconsin-Madison, Madison, WI 53706, USA; melmquist@wisc.edu (M.E.); audra.sterling@wisc.edu (A.S.); 2 Department of Communication Sciences and Disorders, University of Wisconsin-Madison, Madison, WI 53706, USA; cschabes@wisc.edu; 3 Department of Speech-Language-Hearing Sciences, University of Minnesota-Twin Cities, Minneapolis, MN 55455, USA; korne035@umn.edu (M.K.); finestac@umn.edu (L.F.)

**Keywords:** Down syndrome, autism, measurement, diagnostics

## Abstract

Children with Down syndrome (DS) show considerable variability in social-communication and cognitive profiles, and a subset meet criteria for co-occurring autism. In the present study, we examined the associations between developmental domains and autistic trait severity in toddlers with DS. Participants included 38 toddlers (*M* = 4.19 years, *SD* = 0.99) who completed a home-based assessment, including measures of language, fine motor, and visual reception skills. Caregivers also completed standardized questionnaires on communication and executive functioning. Multiple regression analyses tested the degree of association between these developmental domains and autistic traits. Fewer words produced fewer gestures, and more impaired fine motor and visual reception scores were significantly associated with higher autism trait severity, whereas executive function domains were not significantly associated. Preliminary findings indicate that variability in language and nonverbal developmental skills contributes to the expression of autism traits in DS, underscoring the need for early, multidomain assessment approaches to support accurate identification and tailored intervention.

## 1. Introduction

Down syndrome (DS) is a genetically based neurodevelopmental condition caused by the presence of a full or partial extra copy of chromosome 21 (Bull, 2020). It is the leading genetic cause of intellectual disability with a prevalence of 1 in every 707 births (Mai et al., 2019). Individuals with DS share a broad developmental phenotype that often includes hypotonia, characteristic craniofacial features, congenital heart conditions, and delays across cognitive, motor, language, and adaptive domains, though substantial variability exists in how these features present (Daunhauer & Fidler, 2011; Martin et al., 2009). This variability is important to consider in early childhood, a pivotal and malleable period of development. Within this profile, relative strengths in nonverbal communication (Iverson et al., 2003; Stefanini et al., 2007) contrast with persistent challenges in expressive language and adaptive functioning (Abbeduto et al., 2007; Martin et al., 2009).

One source of variability is co-occurring autism spectrum disorder (ASD) in DS. ASD is a neurodevelopmental disorder characterized by persistent deficits in social communication and interaction, and restricted, repetitive patterns of behavior, interests, or activities (American Psychiatric Association, 2013). Historically, a co-diagnosis of ASD in DS was considered rare (Attwood et al., 1988; Gibbs & Thorpe, 1983). More recent research has challenged this assumption (Diniz et al., 2022; Spinazzi et al., 2023), with current prevalence estimates ranging between 16 and 42%, depending on characteristics of the sample (e.g., chronological age, non-verbal IQ) and diagnostic measures employed (Oxelgren et al., 2017; Richards et al., 2015).

Accurate identification and diagnosis of ASD in young children with DS can be challenging as some behavioral phenotypes in DS overlap with the core traits of ASD (Bradbury et al., 2021; Capone et al., 2005; Reilly, 2009; Versaci et al., 2021). For instance, individuals with DS without ASD have impairments in expressive language (Abbeduto et al., 2007; Martin et al., 2009), unique social profiles (Næss et al., 2016; van Gameren-Oosterom et al., 2011), and restricted interests (Evans et al., 2014; Evans & Gray, 2000; Reilly, 2009)—all features that resemble autistic traits. This phenotypic overlap is particularly pronounced in early childhood, when developmental delays can obscure or mimic the core features of ASD. As a result, ASD is often diagnosed later in DS (Reilly, 2009; Versaci et al., 2021).

Understanding how autistic traits emerge and vary within DS is critical because these traits are not expressed uniformly and may interact differently with the broader DS phenotype. When autistic traits are recognized early, interventions can be individualized to address both shared and distinct needs of children with DS and co-occurring DS and ASD (DS+ASD), ultimately supporting gains in language, social interaction, academic learning, and adaptive functioning (Bradbury et al., 2021; Versaci et al., 2021). Despite growing recognition of the co-occurrence of ASD in DS, relatively few studies have examined how autistic traits present and vary within DS. The present study addresses this gap by examining autistic traits among young children with DS and exploring how these traits relate to key developmental domains, including language, executive functioning, and broader developmental skills.

### 1.1. Language Development in DS and Autism

Language development is one of the most prominent and well-documented areas of difficulty for individuals with DS (Abbeduto et al., 2007; Martin et al., 2009). In early development, children with DS exhibit delays in speech and language milestones, with expressive language particularly affected. These expressive challenges include smaller vocabularies (Berglund et al., 2001; Del Hoyo Soriano et al., 2022), limited use of grammatical morphemes (Finestack & Abbeduto, 2010; Price et al., 2008), and reduced utterance length (Kover et al., 2012; Price et al., 2008). These expressive language impairments are further influenced by decreased speech intelligibility stemming from both anatomical and motor-speech factors (Roberts et al., 2007; Stoel-Gammon, 2001). In contrast, receptive vocabulary is often described as a relative strength, with children typically understanding more words than they can produce (Abbeduto et al., 2007). However, receptive vocabulary still develops at a slower rate compared to neurotypical peers and may plateau over time, suggesting broader limitations in lexical learning and retention (Laws & Gunn, 2004; Loveall et al., 2016).

When ASD co-occurs with DS, language impairments are often more pronounced. Studies consistently show that children with DS+ASD have lower expressive language (Cook et al., 2021; Dimachkie Nunnally et al., 2021) and receptive language abilities (Hamner et al., 2020; Molloy et al., 2009) than peers with DS-only. These findings suggest that co-occurring autism may exacerbate language vulnerabilities in DS or alter developmental trajectories that are characteristic of the DS phenotype, leading to increased variability in communication outcomes.

In addition to these language-related challenges, ASD also introduces differences in social interaction that are conceptually distinct from language delays. These include reduced reciprocity, diminished shared affect, and differences in the social use of nonverbal communication (American Psychiatric Association, 2013). Gesture use is one area where social-interaction differences become especially evident. In DS, gesture use is often considered a relative strength and supports early communication, joint attention, and word learning (Caselli et al., 1998; Iverson et al., 2003; Stefanini et al., 2007). However, when autistic traits are present, it is not only the quantity but also the social function of gestures that changes. In toddlers with DS, elevated autistic traits have been linked to fewer gestures, including reduced social use of gestures during early communication (La Valle et al., 2025). Broader work spanning early childhood through young adulthood (age range: 0–21 years) has similarly shown that individuals with DS+autism use fewer gestures and with more limited diversity compared to peers with DS-only (Cook et al., 2021). Furthermore, increases in autistic traits have been found to be associated with a decrease in the frequency of gesture use in DS (age range: 9–22 years; Lorang et al., 2017). These findings reflect both language-related vulnerabilities and broader ASD-related differences in social interaction.

Expressive vocabulary, receptive vocabulary, and gesture use capture key components of early communication that appear sensitive to the presence of autistic traits in DS. Distinguishing language-specific challenges from social-interaction differences can provide a clearer understanding of how ASD features intersect with the DS phenotype and help identify which aspects of communication are most closely associated with autistic traits in young children with DS.

### 1.2. Executive Functions in DS and Autism

Executive functions (EFs) refer to a set of cognitive abilities that support goal-directed behavior and involve the control of information processing (Marcovitch & Zelazo, 2009; Zelazo & Müller, 2002). These processes support the ability to hold and manipulate information, inhibit automatic or competing responses, flexibly shift between tasks or mental sets, and plan and organize behavior across time (Marcovitch & Zelazo, 2009; Zelazo & Müller, 2002). In neurotypical toddlers, these abilities are only beginning to emerge, with early growth typically seen first in simple inhibitory control and working memory tasks, followed by gradual improvements in cognitive flexibility and planning during the preschool years (Zelazo & Müller, 2002). Because EF skills develop in a stepwise fashion in early childhood, even subtle delays or differences may meaningfully alter developmental trajectories (Best et al., 2009).

In young children with DS, EFs are an area of ongoing investigation with mixed findings across studies (see Tungate & Conners, 2021, for a review). Some studies suggest relative strengths in certain EF components, such as cognitive flexibility and attention shifting (Daunhauer et al., 2014; Lanfranchi et al., 2010; Lee et al., 2011), while inhibitory control and working memory are more impaired (Daunhauer et al., 2014; Lee et al., 2011). Because these EFs are just beginning to develop in early childhood, differences in DS may appear subtle or context-dependent, with some difficulties emerging only during challenging tasks or transitions (Daunhauer et al., 2014). Taken together, findings indicate that early EF development in DS may follow a similar developmental sequence to that of neurotypical children, but with greater vulnerability in foundational skills, such as working memory and inhibitory control.

In autism, EF challenges are more consistently reported than in DS. Though impairments in EFs are not a core diagnostic feature of ASD, studies have found that young autistic children may show challenges in inhibitory control (Christ et al., 2011; Pellicano et al., 2017), cognitive flexibility (Pellicano, 2010; Semrud-Clikeman et al., 2014), working memory (Joseph et al., 2005; Pellicano et al., 2017), and planning (Diamond, 2013; Pellicano, 2010). However, autistic individuals are heterogeneous, with studies reporting that roughly 30–70% of autistic children show challenges in EF tasks (Pellicano, 2010). When viewed developmentally, autistic toddlers often show early differences in foundational EF skills, such as inhibitory control and working memory, similar to patterns observed in DS. They also tend to exhibit earlier and more consistent difficulties in cognitive flexibility and planning than would be expected based on typical developmental trajectories. These patterns suggest both shared vulnerabilities across groups and EF profiles that may be more characteristic of autism.

Far less is known about EFs in children with co-occurring DS and autism. Existing work on DS+autism has primarily examined social (e.g., Channell et al., 2015; Godfrey et al., 2019), behavioral (e.g., Bradbury et al., 2021; Godfrey et al., 2019; Hamner et al., 2020), and adaptive features (e.g., Bradbury et al., 2021; Molloy et al., 2009), leaving EFs relatively unexplored. However, one study conducted by Pritchard et al. (2015) examined the patterns of EF strengths and weaknesses in children with DS and co-occurring conditions, such as autism. Their results indicated that children with DS+autism had significantly lower scores on the Emergent Metacognition Index (reflecting working memory and planning/organization) on the Behavior Rating Inventory of Executive Function–Preschool Version (BRIEF-P; Gioia et al., 2003) compared to both DS-only and co-occurring DS and Disruptive Behavior Disorder groups. This suggests that when autism co-occurs with DS, weaknesses in specific EF domains may be more evident. These findings provide preliminary evidence that EFs could be an aspect of development where vulnerabilities in DS (e.g., inhibitory control, working memory) and autism (e.g., flexibility, planning) converge or interact. Examining EFs in young children with DS provides an opportunity to identify early markers of co-occurring autism and understand how cognitive control processes shape emerging self-regulation and adaptive functioning in this population.

### 1.3. Broader Developmental Domains and Autism Traits

Beyond language and EFs, broader developmental domains, including fine motor skills and visual reception, may also influence how autism traits manifest in children with DS. Fine motor development in DS shows considerable variability, with some children acquiring skills along trajectories that are only mildly delayed relative to neurotypical peers, and others showing more pronounced differences in coordination, precision, and timing (Needham et al., 2021). These differences are linked to hypotonia, ligamentous laxity, and motor planning difficulties (Harris, 1984; Palisano et al., 2001). In children with DS+autism, fine motor difficulties may be more evident, often manifesting as challenges in praxis and imitation (Fournier et al., 2010; Godfrey et al., 2019). Such differences can, in turn, limit opportunities for play, exploration, and early social engagement (Iverson, 2010).

Further, visual reception and visuospatial processing show distinctive, but variable patterns in DS. While some aspects of visual-spatial reasoning are relative strengths compared to verbal skills, tasks that require fine-grained visual discrimination or integration of visual–motor information often reveal weaknesses (D. Fidler et al., 2006; Yang et al., 2014). These skills are foundational for attending to and interpreting information in the environment, such as tracking objects, recognizing faces, and integrating visual input with motor actions during play (Karmiloff-Smith et al., 2012; Yang et al., 2014). Differences in visual reception may therefore influence how children attend to social cues or navigate shared activities (Mundy & Newell, 2007). For children with DS+autism, these patterns may be even more variable, with greater difficulties in shifting visual attention and processing socially meaningful visual information, such as eye gaze or facial expressions (Channell, 2020; Karmiloff-Smith et al., 2012). Such challenges may contribute to reduced social orienting and joint attention, hallmark features of autism (Mundy & Newell, 2007).

Collectively, these domains provide critical context for understanding the expression of autistic traits in DS. Lower visual reception and fine motor abilities may amplify or obscure autism-specific behaviors, highlighting the need for nuanced assessment approaches that consider both shared and syndrome-specific developmental patterns.

## 2. Research Questions

Previous research has shown that autistic traits occur at elevated rates in children with DS (e.g., Bradbury et al., 2021; Spinazzi et al., 2023). To better understand co-occurring autism in DS, it is critical to examine how autistic traits relate to key developmental domains, including language and EFs. Therefore, we had the following research questions:

1. How are early communication skills (words understood, words produced, and gesture use) associated with autistic traits in toddlers with DS?
  - We predicted that reduced gesture production and a smaller number of words produced and understood would be associated with higher autistic traits, as difficulties in these early communication components have been linked to differences in social engagement and delayed language acquisition in young autistic children (Luyster et al., 2008; Wetherby et al., 2004).
2. How are components of executive functioning (inhibit, shift, emotional control, working memory, plan/organize) associated with autistic traits in toddlers with DS?
  - We hypothesized that challenges in inhibitory control and working memory would be associated with higher autistic traits, as difficulties in these EF components have been linked to impaired social communication, self-regulation, and adaptive behavior (Christ et al., 2011; Daunhauer et al., 2014).
3. How are other developmental domains (fine motor and visual reception) associated with autistic traits in toddlers with DS?
  - Fine motor and visual reception abilities were expected to be associated with higher autistic traits. Difficulties in these developmental domains have been linked to reduced adaptive functioning, slower learning trajectories, and greater social-communication challenges (Bradbury et al., 2021; Godfrey et al., 2019).

## 3. Method

### Participants

Participants included 38 individuals with DS (*M* (*SD*) = 4.19 (0.99), range = 2.24–6.04 years). Although children ages 2;0–5;11 at the time of consent were eligible, the enrolled sample skewed slightly older, yielding a mean age of 4.19 years. The sample included 22 females, 15 males, and one participant for whom sex was not reported. Data came from a larger multi-site study designed to assess the reliability and validity of language measures for young children with DS. Inclusion criteria of the larger study were: (a) children were between 2;0 and 5;11 years of age at the time of consent, (b) English was the primary language spoken in the home, (c) no evidence of a serious, uncorrected visual impairment confirmed through parent report, (d) no more than a severe hearing loss confirmed through parent report, (e) diagnosis of trisomy 21 or translocation confirmed through genetic testing; and (f) primary caregiver(s) were willing to participate in the study. Parents provided confirmation of a medical diagnosis of DS. To be included in the current study, participants were required to have complete data on the measures analyzed for the current study that were collected during the home visit. Participants were recruited from clinics, centers, and early intervention providers from two midwest cities in the United States and their surrounding areas.

The race of the 38 children was reported by caregivers as: 32 White, three Black or African American, one Asian, one American Indian or Alaska Native and White, and one unknown, due to missing data. Additionally, 35 of the participants were non-Hispanic, one participant was Hispanic, and two were missing ethnicity data. One parent noted that their child had a co-occurring autism diagnosis. See Table 1 for an overview of child demographics.

## 4. Measures

Study procedures were approved by the primary site’s institutional review board (IRB # 2023-0927) at the University of Wisconsin-Madison. Caregivers provided consent. Study procedures took place over the course of four weeks. The larger study included caregiver self-collected videos and audio recordings that were obtained within two weeks before and after a home visit from the research team. During the 2 to 3 h home visit, trained examiners administered the Mullen Scales of Early Learning (MSEL; Mullen, 1995) and the Preschool Language Scales, Fifth Edition (PLS-5; Zimmerman et al., 2011). They also collected a 15 min play-based examiner-child language sample. Examiners also completed the Childhood Autism Rating Scale, 2nd edition (CARS-2; Schopler et al., 2010). During the home visit, caregivers completed a family background demographic survey, and questionnaires related to their own mental health, their child’s communication and intelligibility, and their child’s EFs. The current study focuses on data collected during the home-visit, including examiner-rated autistic traits (CARS-2), caregiver-reported number of words understood and produced and gesture use (MacArthur-Bates Communicative Development Inventory, Words and Gestures [MCDI-WG]; Fenson et al., 2023), caregiver-reported executive functioning, and examiner-administered measures of fine motor and visual reception skills.

### 4.1. Childhood Autism Rating Scale, Standard Version

The CARS-2, Standard Version (Schopler et al., 2010) is a 15-item rating scale that quantifies the presence of autistic traits. An examiner completes the scale based on direct observation and interaction with the child, assigning ratings from one to four for each item, with increments of 0.5. Lower scores reflect minimal or no autistic traits, while higher scores reflect more autistic traits. For children under 13 years of age, a total score of 28 or higher is used as the threshold for an autism classification on the CARS-2. However, in the present study, scores were used continuously to characterize variability in autistic traits rather than for diagnostic classification.

Examiners across both data collection sites established 80% reliability within one point of a gold-standard score (provided by an expert CARS-2 rater) across three consecutive videos of children with DS before scoring participant data. Raters used observations from the entire visit to score the CARS-2. When rating expressive communication items, examiners considered any intentional communicative act, including speech, sign, gesture, or other augmentative forms, as verbal communication, reflecting the multimodal communication profiles typical in DS. Cross-site reliability was assessed through agreement within one point after a secondary rater watched 30 min of the MSEL and the examiner-child play interaction. This procedure was completed on 20% of the files. Ninety one percent of the files agreed within one point across the 15 items.

### 4.2. MacArthur-Bates Communicative Development Inventory, Words and Gestures (MCDI-WG)

The MCDI-WG, Third Edition (Fenson et al., 2023) is a caregiver-reported questionnaire that captures early vocabulary and gesture use. The MCDI-WG includes single words (e.g., “ball,” “down”) and common actions and gestures (e.g., peekaboo, push toy car) typically used by American English–speaking children between 8 and 18 months of age. The MCDI-WG form was selected given that children with DS are often delayed in spoken language development and may otherwise demonstrate floor effects on more advanced forms (Berglund et al., 2001; Esbensen et al., 2017). Caregivers indicated whether their child understood, produced, or understood and produced each word. Because many children with DS rely on signs or augmented and alternative communication, caregivers were asked to report whether their child produced a word through these modalities. Caregivers completed the MCDI-WG during the home visit. The following raw scores were used in analyses: Words Understood, Words Produced, and Total Gestures. We used raw scores because the age range for normative data on the MCDI-WG (i.e., percentiles) is for children aged 8 to 18 months, and our participants were older than 18 months.

### 4.3. Behavior Rating Inventory of Executive Function, Preschool (BRIEF-P)

The BRIEF-P (Gioia et al., 2003) is a caregiver- or teacher-reported questionnaire that measures EFs in children aged 2 to 6 years. Caregivers completed the BRIEF-P during the home visit. The BRIEF-P consists of 63 items that yield raw scores across five domains: Inhibition, Emotional Control, Shift, Working Memory, and Plan/Organize. We used raw scores in our analyses because we had one participant who was slightly older than the standardized age range, preventing use of standardized scores for all participants.

### 4.4. Mullen Scales of Early Learning (MSEL)

The MSEL (Mullen, 1995) is a standardized play-based developmental assessment for children aged birth to 5 years, 8 months. Four subscales were administered by trained examiners: Expressive Language, Receptive Language, Fine Motor, and Visual Reception. For analyses, only raw scores from Fine Motor and Visual Reception subscales were included. Raw scores were selected to minimize floor effects and maximize sensitivity, given that standardized scores often lack appropriate norms for children with DS (Esbensen et al., 2017). Specifically, for Visual Reception, 31 participants were at floor, two were below floor and for two participants, we were not able to calculate *t*-scores. Three participants scored above floor (*M* = 29, *SD* = 5.35, range: 22–35). For Fine Motor, 35 participants were at floor; for two participants, we were not able to calculate *t*-scores. One participant scored above floor (*t*-score = 30).

### 4.5. Data Analysis

All data analyses were conducted using the R statistical computing environment (Version 4.5.1; R Core Team, 2025). Before formal analyses, we conducted a series of preliminary analyses. Examination of data distributions revealed that most variables were non-normally distributed (skewness: −0.68 to 1.51; excess kurtosis: 1.91 to 5.47), prompting the use of non-parametric methods for subsequent preliminary analyses. Given the multi-site nature of the study, we used Wilcoxon rank-sum tests with continuity corrections to assess whether key study variables differed across sites, accounting for potential variation in recruitment, participant characteristics, and assessment procedures. No significant site differences emerged for study variables except for CARS-2 scores (W = 101.5, *p* = 0.035, effect size *r* = 0.34). We attribute this difference not to administrative inconsistencies—given the previously established between-site reliability of CARS-2 scores (91%)—but rather to site-level variation in participants’ autistic traits. However, because CARS-2 was the primary outcome variable, Site was included as a covariate in all planned analyses to account for potential site-related confounds. Furthermore, we elected to keep Site as a covariate in our final models regardless of significance level. Although the 2- to 5-year age range is commonly used in studies of early language development in DS (e.g., Parikh & Mastergeorge, 2018; Lorang et al., 2018), we used Spearman’s rank correlation to assess associations between chronological age and study measures, given our use of raw scores in the analyses. Age was significantly correlated with the following measures: Words Produced (ρ = 0.39, *p* = 0.018), Visual Reception (ρ = 0.62, *p* < 0.001), Fine Motor (ρ = 0.49, *p* = 0.002), Inhibition (ρ = 0.39, *p* = 0.014), Working Memory (ρ = 0.37, *p* = 0.021), and Shifting (ρ = 0.45, *p* = 0.004); Age was therefore included as a covariate in models involving these variables. Similarly to Site, Age was kept in all final models regardless of significance level.

Given the exploratory nature of our study, we aimed to examine how individual components within each developmental domain (e.g., RQ1 communication: receptive, verbal expressive, and nonverbal expressive language) were related to autistic traits; therefore, we conducted a series of regression analyses. To minimize the impact of underpowered models given our sample size, we ran separate models for each study measure. Diagnostic plots from ordinary least squares (OLS) regression models indicated mild heteroscedasticity and a small number of moderately influential observations (*n* = 1–4 across all models). To mitigate the impact of outliers and improve inference robustness, we employed robust linear regression using MM or S-estimation with bi-square weighting. Model results were consistent with those obtained via OLS, but we report findings from the robust regression models. Robust regression analyses were conducted using the robustbase package (version 0.99-6, Maechler et al., 2025). CARS-2 scores were used as the outcome variable in all models. Despite the use of robust regressions, our parameter estimates may be unstable, given our sample size, and results should be interpreted accordingly. To address inflation of Type I errors due to multiple comparisons, Bonferroni corrections were applied at the hypothesis level (i.e., each research question) based on the number of variables related to our hypotheses (i.e., we did not apply corrections to Intercept, Site, and Age).

### 4.6. Missing Data

We did not have any missing data across the measures used in our regression analyses. However, we did have some missing data related to participant demographics. Specifically, two participants did not report sex, race, and ethnicity. For these variables, we have a “not-reported” category.

#### Positionality and Language Use

In alignment with neurodiversity-affirming practices and community preferences within the autism field (Bottema-Beutel et al., 2021), we use identity-first language (e.g., “autistic traits,” “autistic individuals”) throughout this manuscript. Our research team consists of clinicians and researchers with extensive experience working with neurodivergent children and families, and we approach this work from a strengths-based, non-pathologizing perspective. Our goal is to describe developmental variability among toddlers with Down syndrome without reinforcing deficit-based interpretations or stigma.

## 5. Results

### 5.1. RQ1: Associations Between CARS-2 Scores and the MCDI-WG

**Figure 1:**
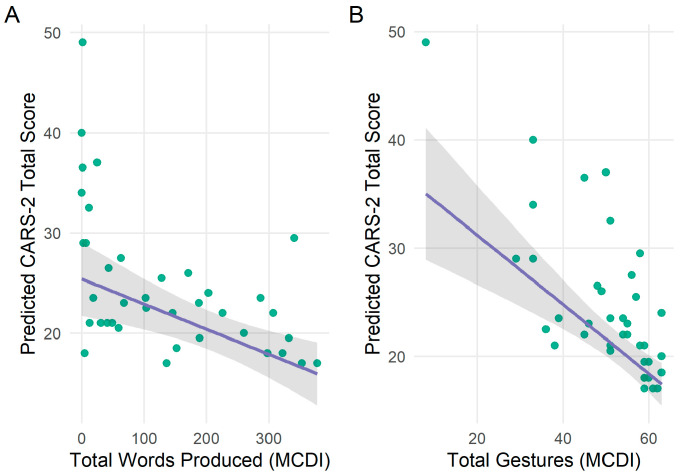
(**A**)Associations between total words produced and autistic traits in toddlers with Down syndrome. (**B**) Associations between total gestures and autistic traits in toddlers with Down syndrome.

We ran three robust regression models to evaluate verbal and non-verbal language predictors of autistic traits, as assessed by the CARS-2 (higher scores reflect greater severity), using the MCDI-WG (see Table 2 for full model results). After controlling for chronological Age and Site, Words Produced significantly predicted autistic traits in toddlers with DS (*b* = −0.03, *SE* = 0.01, *t*(34) = −3.43, *p* = 0.006). Specifically, each additional word produced was associated with a 0.33-point reduction in CARS-2 scores, such that every 33 words corresponded to a one-point decrease in CARS-2 scores. The number of gestures produced was significantly associated with autistic traits (*b* = −0.30, *SE* = 0.08, *t*(35) = −3.86, *p* = 0.003). When controlling for Site specifically, CARS-2 scores decreased by approximately 0.30 points for each additional gesture produced; this resulted in a one-point reduction in CARS-2 scores for every three to four additional gestures reported by caregivers. In contrast, the number of words understood was not significantly associated with autistic traits in toddlers with DS (*b* = −0.01, SE = 0.01, *t*(35) = −1.16, *p* = 0.759). Please see Figure 1 for associations between CARS-2 scores and the MCDI-WG.

### 5.2. RQ 2: Associations Between CARS-2 and BRIEF-P

To address our second research question, using raw scores from the BRIEF-P, we ran five robust regression models to examine the associations between components of EFs (i.e., Inhibit, Shift, Emotional Control, Working Memory, and Plan/Organize) and autistic traits in toddlers with DS, as assessed by the CARS-2 (see Table 3 for full model results). Across all our models, after controlling for Site and, when applicable, chronological Age, we found no significant associations between EFs and autistic traits in toddlers with DS.

### 5.3. RQ 3: Associations Between CARS-2 and MSEL

**Figure 2:**
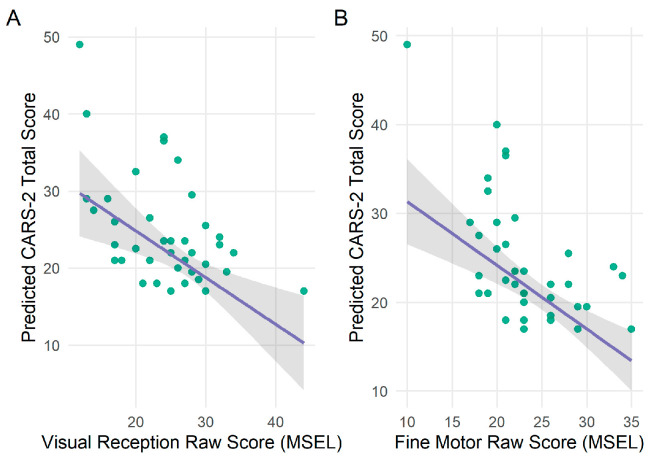
(**A**) Associations between visual reception raw scores and autistic traits in toddlers with Down syndrome; (**B**) Associations between fine motor raw scores and autistic traits in toddlers with Down syndrome.

To address our final research question, using raw scores from the MSEL, we ran two robust regression models to examine the associations between Fine Motor, Visual Reception and autistic traits (i.e., CARS-2 scores) in toddlers with DS (See Table 4). After controlling for child chronological Age and Site, Visual Reception (*b* = −0.61, *SE* = 0.17, *t*(34) = −3.48, *p* = 0.002) and Fine Motor (*b* = −0.72, *SE* = 0.15, *t*(34) = −4.76, *p* < 0.001) were significantly associated with autistic traits in toddlers with DS. Specifically, a 1-point increase in visual reception raw scores was associated with a 0.61 reduction in CARS-2 scores, or conversely, roughly each additional 1.6 points on the visual reception subscale was associated with a 1-point reduction on the CARS-2. Similarly, we found that each additional raw score on the fine motor subscale was associated with a 0.72 reduction in CARS-2 scores, or for approximately each additional 1.4 points in the fine motor score was associated with a 1-point reduction on the CARS-2. Please see Figure 2 for associations between CARS-2 and MSEL.

## 6. Discussion

Understanding how early developmental domains are associated with the expression of autistic traits is critical for clarifying the early behavioral phenotype of toddlers with DS+ASD. Although prior work has documented elevated rates of co-occurring ASD in DS (e.g., DiGuiseppi et al., 2010; Spinazzi et al., 2023), less is known about how individual differences in language, EFs, and other developmental skills are associated with autistic traits in DS. The present study addressed this gap by examining how early verbal, nonverbal, and motor abilities were associated with autistic trait severity in toddlers with DS.

### 6.1. Language and Autistic Traits in DS

We examined the association between caregiver-reported child language and autistic traits, using raw scores from the MCDI-WG and total scores from the CARS-2. Our results suggest that there are significant relationships between the words produced and the use of gestures with autistic traits. In contrast, no significant relationship was found between the number of words understood and autistic traits. These findings provide preliminary evidence that expressive vocabulary and gesture use are associated with variability in autistic traits. Notably, gesture use showed a stronger association with autistic traits relative to words produced, such that toddlers who used fewer gestures were rated as having higher levels of autistic traits. This association underscores the social-communicative salience of gestures in early development and highlights their potential as an early behavioral marker of elevated autism traits within DS.

Our results related to gesture use align with prior work identifying gestures as an early marker of social-communication development in autism (Luyster et al., 2008; Wetherby et al., 2004). Gesture use is typically a relative strength in DS (Caselli et al., 1998; Iverson et al., 2003; Stefanini et al., 2007) and supports joint attention and early word learning (Cochet & Byrne, 2016; Liu et al., 2024). Our results extend this literature by suggesting that reduced gesture production is associated with higher levels of autistic traits. Providing preliminary evidence that early differences in nonverbal communication may be closely linked to the expression of autism-related behaviors in DS. Differences in gesture use may reflect variability in social attention, imitation, or motor planning, skills foundational to social interaction and spoken language (Cochet & Byrne, 2016; Liu et al., 2024). Because gestures often serve as a bridge to speech, limited gesture production may constrain opportunities for social learning and expressive language growth (Grieco et al., 2015).

The number of words produced were significantly associated with autistic traits, indicating that, on average, children with smaller expressive vocabularies had higher CARS-2 scores. This finding extends prior work documenting expressive language challenges as a hallmark of DS (Abbeduto et al., 2007; Martin et al., 2009) and suggests that variability in expressive language may contribute to individual differences in autistic trait expression. Expressive vocabulary in DS depends heavily on opportunities for social engagement and successful coordination of motor, cognitive, and social-cognitive processes (Abbeduto et al., 2007). When expressive development is shaped by factors, such as motor speech challenges (Paul et al., 2011; Roberts et al., 2007), reduced initiation of joint attention, or lower social motivation, these influences may amplify the expression of autistic traits (Luyster et al., 2008; Shumway & Wetherby, 2009). Together, these factors likely contribute to the finding that toddlers with fewer words produced exhibited higher levels of autistic traits. Questions remain on whether this pattern reflects areas of overlapping phenotype between DS and autism (e.g., shared challenges in expressive communication) or if it represents features more specific to a DS+ASD subgroup characterized by additive difficulties in social communication. Future work comparing young children with DS-only and DS+autism profiles will be critical for disentangling these possibilities.

In contrast to expressive vocabulary and gesture use, words understood were not significantly associated with autistic traits. This pattern suggests that receptive vocabulary may be less affected by the presence of autistic traits in early development. One possibility is that comprehension remains relatively stable in young children with DS (Abbeduto et al., 2007; Roberts et al., 2007), as comprehension tasks may place fewer demands on social initiation or speech-motor planning (Tager-Flusberg et al., 2005). Alternatively, caregiver-report measures, such as the MCDI-WG may be less sensitive to subtle differences in comprehension or attentional engagement that accompany elevated autistic traits (Ellis Weismer et al., 2010; Luyster et al., 2008). It is also possible that comprehension strengths in DS become more apparent later in development as increased experience and exposure support comprehension of familiar and event-based vocabulary (Chapman et al., 2006; Glenn & Cunningham, 2005). Future research using direct, performance-based assessments of language comprehension could clarify whether the absence of a significant association reflects genuine resilience in comprehension skills or limitations in measurement sensitivity during early childhood.

These preliminary findings underscore the close links between expressive communication and autistic traits in toddlers with DS, while also raising important questions about the interpretation of autistic traits in the context of the DS behavioral phenotype. The overlap between DS- and autism-related features is particularly relevant for language, as both groups often show delays in expressive development, reduced spontaneous communication, and challenges in social reciprocity (Abbeduto et al., 2007; Luyster et al., 2008; Roberts et al., 2007). Given that several CARS-2 items capture language and cognitive abilities, higher autism scores in DS may, in part, reflect this shared developmental profile rather than autism-specific social impairments. This measurement overlap makes it difficult to disentangle whether elevated scores represent true co-occurring autism or the additive impact of DS-related expressive and cognitive challenges. Future research incorporating multiple diagnostic tools and group comparisons (e.g., DS-only vs. DS+autism) will be critical for clarifying how language and cognitive domains contribute to CARS-2 classifications and for refining how autistic traits are measured within DS.

### 6.2. Executive Functions and Autism Traits in DS

We also examined the relationship between EFs and autistic traits, using raw scores from the BRIEF-P. In contrast to our language findings, regression analyses showed that none of the five EF domains (i.e., Inhibit, Shift, Emotional Control, Working Memory, and Plan/Organize) were associated with autistic traits after controlling for site and, when relevant, chronological age (included for Inhibit, Shift, Working Memory). These exploratory analyses suggest that, within this sample of young children with DS, parent-reported EF difficulties were not associated with autistic traits.

Prior work with older children has reported EF challenges in DS+autism, particularly in working memory and planning/organization (Pritchard et al., 2015). Our findings did not reveal significant associations between autistic traits and any EF domains in young children with DS. One explanation for these differing results is developmental timing. The Pritchard et al. (2015) sample included children 3 to 13 years, when higher-order EF processes, such as planning and organization, are more established (Best et al., 2009) and thus, more observable in everyday behavior. In early childhood, EF skills are still emerging (Best et al., 2009; Daunhauer et al., 2014), and caregivers may have difficulty distinguishing autism-related EF differences from the variability expected during the early development. These findings suggest that relationships between autistic traits and EFs may appear later in development as cognitive control demands increase and EF abilities become more differentiated (Daunhauer et al., 2014).

Further, the absence of significant associations may have reflected measurement limitations. The BRIEF-P provides valuable insight into EF behaviors in naturalistic contexts but relies on caregiver perceptions, which may be influenced by general developmental expectations for children with DS (Daunhauer et al., 2014; Isquith et al., 2005). EF-related challenges (e.g., difficulties with inhibition or shifting) may be viewed by parents as normative within DS (Daunhauer et al., 2014), potentially obscuring patterns that overlap with autism traits. Future work incorporating both caregiver ratings and direct, performance-based EF tasks could clarify whether EF weaknesses in toddlers with DS represent later-emerging markers of autism traits or reflect domain-general cognitive characteristics of DS.

### 6.3. Other Developmental Domains and Autism Traits in DS

In our final research question, we examined whether fine motor and visual reception were associated with higher autistic traits in young children with DS, using raw scores from the MSEL. We found that, on average, lower scores on the Visual Reception and Fine Motor subscales were significantly associated with higher levels of autistic traits. This finding suggests that autism-related differences in DS likely extend beyond social-communication to broader developmental domains, such as aspects of cognition and motor development.

The association between visual reception skills and autistic traits suggests that, on average, young children with DS with weaker visual processing and attention abilities show elevated autistic traits. Visual reception tasks require sustained attention, visual discrimination, and problem-solving skills that underlie joint attention, imitation, and symbolic understanding (Landry & Bryson, 2004; Mundy & Newell, 2007). Thus, lower visual reception scores may reflect challenges in visual processing or attentional control that limit children’s ability to efficiently process social and environmental cues (Chawarska et al., 2013; Keehn et al., 2013). Over time, these visual-attentional differences may constrain opportunities for social learning and contribute to the heterogeneity observed in autism traits among children with DS.

Similarly, on average, lower fine motor scores were significantly associated with higher autistic traits, underscoring the close links between motor coordination and social-communicative development. Fine motor abilities support gesture production, object exploration, and imitation, which in turn facilitate expressive communication and early cognitive growth (Iverson, 2010; Libertus & Hauf, 2017). Difficulties with motor planning or coordination may reduce the frequency and quality of socially oriented actions, limiting opportunities for interaction and feedback from caregivers. Although motor delays are characteristic of DS (Palisano et al., 2001; Pereira et al., 2013), these findings suggest that weaker fine motor abilities may further contribute to variability in the expression of autistic traits within DS.

In summary, these findings highlight the contribution of visual reception and fine motor abilities to variability in autistic traits among young children with DS. Early differences in visual reception and motor coordination were associated with higher levels of autistic traits in this sample. These associations may reflect overlapping areas of developmental vulnerability, but longitudinal data are needed to determine the nature or direction of these relations. Future work should explore whether interventions that strengthen attentional and motor skills can promote social-communicative and developmental growth in this population.

### 6.4. Study Limitations and Future Directions

Several limitations should be considered when interpreting the findings of this study. First, although the sample size was consistent with other research on co-occurring autistic traits in DS (e.g., Bradbury et al., 2021; Channell et al., 2015; La Valle et al., 2025), it was relatively small, which limits statistical power, stability of parameter estimates, and generalizability. As a result, our results should be interpreted accordingly, especially given the number of analyses completed. Similarly, while we opted to conduct more regression models with fewer predictors to reduce overfitting and underpowered models, given our sample size, parameter estimates may still be unstable, and our results should be interpreted accordingly. Future work should include larger and more diverse samples to confirm these associations and explore potential moderators, such as cognitive ability, sex, or socioeconomic factors. Second, while our regression models allowed us to examine the direction of associations, the cross-sectional design precludes any causal inferences or conclusions about developmental change. As such, these findings should be interpreted as exploratory rather than indicative of developmental mechanisms. Longitudinal data are needed to determine whether early differences in language, executive functioning, and other developmental domains prospectively predict changes in autism symptomatology over time.

Third, the racial and ethnic composition of our sample was predominantly White (32 of 38 participants), which limits the generalizability of these findings to more diverse populations. Communication behaviors, caregiver-report ratings, and the interpretation of autism-related traits can vary across cultural and linguistic contexts. Therefore, caution is warranted when applying these results to children from historically underrepresented communities. Future research that includes more racially and culturally diverse samples will be essential for improving the representativeness and applicability of this work.

Additionally, autistic traits were documented using the CARS-2, a widely used diagnostic tool developed and validated for identifying idiopathic autism (DiGuiseppi et al., 2010; Diniz et al., 2022). Therefore, the CARS-2 may not capture the full range of subtle social-communication differences present in DS. Incorporating multiple assessment methods, including caregiver interviews, standardized diagnostic instruments, and direct behavioral measures, would provide a more comprehensive understanding of autistic-related traits in this population. Further, parent-report measures of language and executive functioning, including the MCDI-WG (Fenson et al., 2023) and the BRIEF-P (Gioia et al., 2003), may be influenced by caregivers’ expectations and experiences with the DS phenotype (Daunhauer et al., 2014; Isquith et al., 2005). However, these instruments were selected because they provide ecologically valid information about children’s everyday communication and behavior across settings that are often difficult to capture during a single structured assessment. Future work should examine whether the relationships between developmental domains and autistic traits differ based on assessment method (e.g., caregiver-report vs. standardized assessment).

Another limitation of the present study is the absence of a DS-only comparison group or a clinically confirmed DS+ASD subgroup. Because our analyses relied on continuous ratings of autistic traits within a single DS sample, the study cannot determine whether the observed associations reflect autism-specific characteristics or variability inherent to the DS phenotype. As a result, these findings should be interpreted cautiously and viewed as hypothesis-generating rather than as evidence of a distinct DS+ASD developmental profile. Future work that includes DS-only and DS+ASD comparison groups, supported by comprehensive diagnostic evaluations, will be critical for distinguishing DS-related developmental patterns from characteristics uniquely associated with co-occurring autism.

Lastly, the current study focused on developmental domains, such as language, EFs, fine motor, and visual reception, other factors likely contribute to autistic traits in DS. Future work should examine the contribution of social motivation, pragmatic language, and joint attention to the developmental pathways linking DS and autism. Longitudinal designs using a combination of caregiver reported and experimental measures will be particularly valuable for identifying early predictors of co-occurring autism and for informing tailored intervention approaches that leverage strengths in social engagement and learning within the DS phenotype.

## 7. Summary and Conclusions

The present findings deepen our understanding of how early developmental domains contribute to variability in autistic traits in young children with DS. The number of words produced, gesture use, fine motor, and visual reception skills were significantly associated with autistic traits, underscoring the multifaceted developmental underpinnings of autistic-related characteristics in DS. These preliminary findings highlight the value of examining cross-domain relationships to better understand early variability in social-communication development. Continued longitudinal, multimethod research will be essential for identifying early developmental predictors of co-occurring autism and for designing targeted interventions that build on individual strengths across developmental domains to support optimal communication outcomes.

## Figures and Tables

**Table 1:** Child Demographics (N = 38).

Variable	M(SD) Unless Otherwise Noted	Range
Chronological age in years	4.19 (0.99)	2.24–6.04
Sex n (%)		
Female	22 (58%)	
Male	15 (39%)	
Missing data	1 (2.63%)	
Race n (%)		
Asian	1 (2.5%)	
Black or African American	3 (7.9%)	
White	32 (85.0%)	
American Indian or Alaska Native and White	1 (2.5%)	
Missing data	1 (2.6%)	
Ethnicity n (%)		
Hispanic or Latino	1 (2.5%)	
Not Hispanic or Latino	35 (92.5%)	
Missing data	2 (5.3%)	
Household income		
Less than $25,000	2 (5.3%)	
$25,000–50,000	3 (7.9%)	
$50,000–75,000	4 (10.5%)	
$75,000–100,000	7 (18.4%)	
$100,000–150,000	9 (23.7%)	
$150,000–250,000	7 (18.4%)	
More than $250,000	6 (15.8%)	
Highest level of education of caregiver who completed the form (n%)		
Graduated high school/GED	3 (7.9%)	
Some college or technical school	4 (10.5%)	
Graduated with an associates/technical college degree	4 (10.5%)	
Graduated college with a bachelor’s degree	11 (28.9%)	
Some graduate work	2 (5.3%)	
Master’s/other graduate degree	14 (36.8%)	
CARS-2	24.68 (7.11)	17–49
MCDI-WG		
Words Produced	133.32 (124.01)	0–378
Words Understood	260.53 (119.90)	37–395
Gesture Use	50.26 (11.78)	0–63
BRIEF-P		
Inhibit	27.39 (7.28)	16–46
Shift	15.68 (4.16)	10–26
Emotional Control	15.26 (4.12)	10–29
Plan/Organize	17.95 (4.73)	10–28
Working Memory	30.74 (6.92)	18–48
MSEL		
Fine Motor	23.13 (5.00)	10–35
Visual Reception	24.29 (6.70)	12–44

Note. GED = General Equivalency Diploma, CARS-2 = Childhood Autism Rating Scale, Second Edition, MCDI-WG = MacArthur–Bates Communicative Development Inventory–Words and Gestures, BRIEF-P = Behavior Rating Inventory of Executive Function–Preschool Version, MSEL = Mullen Scales of Early Learning.

**Table 2:** Robust Regression Summaries for MCDI-WG measures predicting autistic traits as measured by the CARS-2.

Predictor	b	Std. Error	*t*-Value	95% CI	Unadjusted *p*-Value	Bonferroni Corrected *p*-Value
**Model 1: Words Produced**						
Intercept	19.72	3.86	5.11	[11.86, 27.58]	0.000	-
Words produced	−0.03	0.01	−3.43	[−0.04, −0.01]	0.002	0.006
CA	0.11	0.07	1.58	[−0.03, 0.26]	0.123	-
Site	3.80	1.49	2.55	[0.78, 6.83]	0.015	-
**Model 2: Words Understood**						
Intercept	25.10	3.42	7.35	[18.11, 32.09]	0.000	-
Words Understood	−0.01	0.01	−1.16	[−0.03, 0.01]	0.253	0.759
Site	3.8340	1.94	1.97	[−0.09, 7.76]	0.0565	-
**Model 3: Total Gestures Produced**						
Intercept	36.37	4.16	8.7411	[27.87, 44.87]	0.000	-
Gestures	−0.30	0.08	−3.86	[−0.45, −0.15]	0.001	0.003
Site	2.09	1.53	1.370	[−1.03, 5.21]	0.179	-

Note. MCDI-WG = MacArthur–Bates Communicative Development Inventory–Words and Gestures, CA = Chronological age. Model 1 model statistics: R^2^ (robust) = 0.44, Adjusted R^2^ = 0.39, Robust SE = 3.86. Results were obtained using MM-estimation. One observation was downweighted as an outlier. Model 2 statistics: R^2^ (robust) = 0.23, Adjusted R^2^ = 0.18, Robust SE = 4.44. Results were obtained using MM-estimation. One observation was downweighted as an outlier. Model 3 model statistics: R^2^ (robust) = 0.74, Adjusted R^2^ = 0.73, Robust SE = 3.47. Results obtained using S-estimation. Eight observations were downweighted due to large residuals or high leverage.

**Table 3:** Robust Regression Summaries for BRIEF-P measures predicting autistic traits as measured by the CARS-2.

Predictor	b	Std. Error	*t*-Value	95% CI	Unadjusted *p*-Value	Bonferroni Corrected *p*-Value
**Model 1: Inhibition**						
Intercept	21.55	4.59	4.69	[12.19, 30.91]	0.000	-
Inhibition	−0.01	0.13	−0.09	[−0.27, 0.25]	0.932	1.000
CA	0.01	0.09	0.08	[−0.18, 0.19]	0.939	-
Site	4.37	2.11	2.07	[0.10, 8.64]	0.046	-
**Model 2: Shift**						
Intercept	18.04	5.47	3.30	[7.03, 29.05]	0.002	-
Shift	0.46	0.41	1.11	[−0.38, 1.31]	0.274	1.000
CA	−0.07	0.11	−0.67	[−0.29, 0.15]	0.509	-
Site	4.85	2.18	2.23	[0.42, 9.28]	0.033	-
**Model 3: Working Memory**						
Intercept	19.63	6.18	3.17	[7.07, 32.19]	0.003	-
Working Memory	0.08	0.16	0.53	[−0.23, 0.40]	0.602	1.000
CA	−0.01	0.08	−0.15	[−0.18, 0.15]	0.881	-
Site	4.57	2.20	2.08	[0.12, 9.02]	0.045	-
**Model 4: Plan/Organize**						
Intercept	21.41	7.41	2.89	[6.22, 36.60]	0.007	-
Plan/Organize	0.00	0.44	0.00	[−0.90, 0.90]	0.998	1.000
Site	4.16	2.35	1.77	[−0.64, 8.96]	0.086	-
**Model 5: Emotional Control**						
Intercept	24.72	1.64	15.09	[21.39, 28.06]	0.000	-
Emotional Control	−0.22	0.12	−1.88	[−0.45, 0.02]	0.068	0.340
Site	4.30	1.93	2.23	[0.38, 8.22]	0.033	-

Note. BRIEF-P = Behavior Rating Inventory of Executive Function–Preschool Version, CA = Chronological age. Model 1 model statistics: R^2^ (robust) = 0.15, Adjusted R^2^ = 0.08, Robust SE = 4.91. Results were obtained using MM-estimation. One observation was downweighted as an outlier. Model 2 statistics: R^2^ (robust) = 0.22, Adjusted R^2^ = 0.15, Robust SE = 4.89. Results were obtained using MM-estimation. One observation was downweighted as an outlier. Model 3 model statistics: R^2^ (robust) = 0.17, Adjusted R^2^ = 0.09, Robust SE = 4.90. Results obtained using MM-estimation. One observation was downweighted as an outlier. Model 4 model statistics: R^2^ (robust) = 0.16, Adjusted R^2^ = 0.11, Robust SE = 4.24. Results obtained using MM-estimation. One observation was downweighted as an outlier. Model 5 model statistics: R^2^ (robust) = 0.19, Adjusted R^2^ = 0.15, Robust SE = 4.46. Results obtained using MM-estimation. One observation was downweighted as an outlier.

**Table 4:** Robust Regression Summaries for MSEL measures predicting autistic traits as measured by the CARS-2.

Predictor	b	Std. Error	*t*-Value	95% CI	Unadjusted *p*-Value	Bonferroni Corrected *p*-Value
**Model 1: Visual Reception**						
Intercept	24.80	3.23	7.68	[18.48, 31.12]	0.000	-
Visual Reception	−0.61	0.17	−3.48	[−0.95, −0.27]	0.001	0.002
CA	0.25	0.08	2.89	[0.07, 0.42]	0.007	-
Site	3.89	1.46	2.67	[0.93, 6.85]	0.012	-
**Model 2: Fine motor**						
Intercept	29.39	4.25	6.92	[20.73, 38.06]	0.000	-
Fine motor	−0.72	0.15	−4.76	[−1.02, −0.41]	0.000	0.000
CA	0.18	0.08	2.43	[0.03, 0.33]	0.020	-
Site	4.60	1.56	2.94	[1.40, 7.80]	0.006	-

Note. MSEL = Mullen Scales of Early Learning, CA = Chronological age. Model 1 model statistics: R^2^ (robust) = 0.49, Adjusted R^2^ = 0.44, Robust SE = 3.97 Results were obtained using MM-estimation. One observation was downweighted as an outlier. Model 2 statistics: R^2^ (robust) = 0.50, Adjusted R^2^ = 0.46, Robuest SE = 3.65. Results were obtained using MM-estimation. One observation was downweighted as an outlier.

## Data Availability

The data that support the findings of this study are not publicly available because not all participants provided consent for data sharing. For participants who consented, de-identified data will be made available through the NIH INCLUDE Data Coordinating Center (DCC) following completion of data processing and upload.
